# Ethnicity and prostate cancer: the way to solve the screening problem?

**DOI:** 10.1186/s12916-015-0427-z

**Published:** 2015-08-04

**Authors:** Leonard P. Bokhorst, Monique J. Roobol

**Affiliations:** Department of Urology, Erasmus University Medical Center, Rotterdam, The Netherlands

**Keywords:** Incidence, Mortality, Prostate-specific antigen, Prostatic neoplasms, Race, Risk stratification, Screening

## Abstract

In their analysis in *BMC Medicine*, Lloyd et al. provide individual patient lifetime risks of prostate cancer diagnosis and prostate cancer death stratified by ethnicity. This easy to understand information is helpful for men to decide whether to start prostate-specific antigen testing (i.e. screening). A higher lifetime risk of prostate cancer death in some ethnic groups is not automatically a license to start screening. The potential benefit in the form of reducing metastases and death should still be weighed against the potential risk of over diagnosis. In case of ethnicity, this harm-to-benefit ratio does not differ between groups. Stratifying men for screening based on ethnicity is therefore not optimal and will not solve the current screening problem. Other methods for risk-stratifying men have been proven to produce a more optimal harm-to-benefit ratio.

Please see related article: http://www.biomedcentral.com/1741-7015/13/171

## Background

“Prostate cancer is the most common cancer among men, and the second leading cause of cancer death” is perhaps the most frequently used first sentence in any article reporting on prostate cancer today. Although it sounds a bit like a cliché, the true importance of this first statement should not be overlooked. The data on incidence, and especially mortality, were the driving force for many to search for ways to prevent the occurrence of prostate cancer deaths at an early stage. This ultimately culminated (at the end of the previous century) in the start of some of the largest population based screening studies ever conducted [1, 2]. Now, two decades after the start of these trials, we know from the largest trial, the European Randomized study of Screening for Prostate Cancer (ERSPC, [3]), that we are able to reduce the number of men that suffer from metastatic disease by 30 % and men that die of prostate cancer by roughly 20 % on a population-based level [1, 4, 5]. On an individual basis, the prostate cancer mortality reduction can increase up to 51 % when comparing a man choosing to be regularly screened versus a man not screened at all [6]. However, this reduction comes at a considerable cost, i.e. substantial over diagnosis and overtreatment of prostate cancers that were never destined to cause complaints let alone kill (indolent prostate cancer) [1, 7, 8]. On a population-based level, these harms of prostate cancer screening are judged not to outweigh the benefits. Much research is therefore currently done into new markers (e.g. blood, urine, or tissue markers) and technologies (e.g. MRI) to enable the selective detection of aggressive prostate cancers and thereby reduce the harms. However, until truly better markers and technologies become (widely) available, improving the current screening strategies by risk-stratifying men into high risk (and thus potentially a better harm-to-benefit ratio) and low risk (and thus potentially a poorer harm-to-benefit ratio) based on currently available data, seems the best way to go [9]. In addition, instead of offering screening to the entire population, most major guidelines now advise to discuss screening on an individual level [10, 11], starting only if individual potential benefits are judged to weigh against the potential harms of, and by, the individual. In this light, Lloyd et al. [12] recently published in *BMC Medicine* a manuscript that aimed to provide individual men with the so needed easy to grasp lifetime risks of prostate cancer diagnosis and prostate cancer death. They go even further by risk-stratifying men into high and low risk of prostate cancer death, based on race, suggesting a better harm-to-benefit ratio for some ethnicities as opposed to others. Based on the presented UK-based data, they hope to help men make a better informed decision on prostate cancer screening. However, the question is whether there really is a true difference between race and prostate cancer mortality that should trigger screening in the one and not the other, and, if so, is this difference the most optimal way to risk-stratify men for screening?

### Prostate cancer and race

Before discussing these questions in full length, we would like to start by commending the authors for the thorough way the data was analyzed and the usefulness in informing patients of these rather simple looking but straight forward numbers of life time risk. Of course, some remarks on these type of analyses are warranted: ethnicity was missing in some men, errors in linking major databases could have occurred and, perhaps most importantly, the currently measured mortality and incidence data are not related in the sense that the mortality data most likely result from prostate cancer cases diagnosed 10 years earlier, a period in which ethnicity ratios and incidence could have been different. However, overall, the authors did a thorough job, including several sensitivity analyses to look at the effect of imputing different ethnicity rates for the missing data. Generally, the results represent a “best estimate” of the lifetime risk of prostate cancer incidence and mortality and, as said, extremely useful to inform men.

What is of particular interest is the difference in risk of diagnosis and death per race. Black men are roughly at twice the risk of diagnosis and death as compared to white men, who are in turn at roughly twice the risk of diagnosis and death as compared to Asian men [12]. However, the diagnosis-to-death ratio is very similar among all ethnicities. In other words, once diagnosed, there is a one in three chance to die of the disease, irrespective of race. This finding differs from many American studies which not only show a higher risk of diagnosis and death, but also a higher risk of death once diagnosed (i.e. some races present with proportionally more aggressive disease) [13]. As suggested by the authors, this could be based on differences in time of diagnosis (at a more aggressive stage) and differences in treatment based on socioeconomical variances instead of true differences in disease etiology [12]. The reason (either genetic differences or differences in lifestyle) why some races do have a higher occurrence of the disease but once detected do not present with more aggressive disease is interesting and definitively warrants further research. Nevertheless, this finding already has an important implication. The authors suggest that black men in particular should be warned about the risk of prostate cancer death and seek early prostate-specific antigen (PSA) testing (i.e. screening). Indeed, the double lifetime risk of dying from prostate cancer for black men would suggest that the number needed to screen to avoid one prostate cancer death (often used as a measure of screening effectiveness, calculated as the reciprocal of the absolute mortality reduction) would be roughly twice as low as compared to white men. However, based on these data, the number of black men that are diagnosed with a non-lethal prostate cancer will also double. More men will thus be diagnosed and experience the harms of over diagnosis and subsequent overtreatment. In fact, if the lifetime risk of diagnosis and death are both twice as high, the harm-to-benefit ratio of screening will be unchanged. If population-based screening is not deemed ethical on the basis of the currently known harm-to-benefit ratio, is it then ethical to actively promote screening in black men who seem to have a similar harm-to-benefit ratio?

We believe this approach does not adequately address the current conundrum. We should focus in risk-stratifying men based on the best harm-to-benefit ratio. As such, screening should then only be actively offered if the harm-to-benefit ratio strongly favors the benefits and should be requested by the individual and discussed in case of an ambivalent harm-to-benefit ratio, and avoided in men with no benefit, but mostly harms.

Several methods for achieving this better than average harm-to-benefit ratio were studied. Based on modelling data from the ERSPC, limiting screening to specific age groups (i.e. two to three screens between the ages of 55 and 59 years) was shown to maximize the harm-to-benefit ratio [14]. Others suggested to start screening at an even earlier age, stratifying men based on a baseline PSA value, and only actively offer screening in men within the highest 10 % of risk of prostate cancer death [15]. In fact, this strategy was shown to produce a better risk-to-benefit ratio as compared to stratifying men based on race or family history of prostate cancer [16]. Once screening starts, optimizing the harm-to-benefit ratio could be achieved by stratifying men for further, potentially harmful or burdensome testing (e.g. biopsy or an MRI). This has been shown possible by using developed and validated risk calculators [9, 17–19] and is recommended in guidelines (e.g. European Association of Urology).

## Conclusions

Lifetime risks of prostate cancer diagnosis and prostate cancer death specified by race provide patients with useful information on their personal condition and can help in deciding whether to start PSA testing. However, a higher than average risk of prostate cancer death for some groups should still be weighed against the harms of over diagnosis and related overtreatment. Risk stratification on the basis of ethnicity results in a comparable ratio of the harm-to-benefit of prostate cancer screening. Better methods resulting in a more beneficial harm-to-benefit ratio are, however, available and should not be overlooked when considering screening for prostate cancer.

## Abbreviations

ERSPC: European Randomized study of Screening for Prostate Cancer
PSA: Prostate-specific antigen
